# Addressing the Ethical Challenges of Providing Kidney Failure Care for Children: A Global Stance

**DOI:** 10.3389/fped.2022.842783

**Published:** 2022-03-11

**Authors:** Priya Pais, Aaron Wightman

**Affiliations:** ^1^Department of Pediatric Nephrology, St. John's Medical College, St. John's National Academy of Health Sciences, Bangalore, India; ^2^Division of Bioethics and Palliative Care, Department of Pediatrics, University of Washington School of Medicine, Seattle, WA, United States; ^3^Division of Nephrology, Department of Pediatrics, University of Washington School of Medicine, Seattle, WA, United States; ^4^Treuman Katz Center for Pediatric Bioethics, Seattle Children's Research Institute, Seattle, WA, United States

**Keywords:** pediatric kidney failure, ethical challenges, dialysis, kidney transplant, low and middle income countries (LMICs), advocacy, global inequity

## Abstract

Children with kidney failure require kidney replacement therapy (KRT), namely maintenance dialysis and kidney transplant. Adequate kidney failure care consists of KRT or conservative treatment with palliative care. In the context of kidney failure, children depend on parents who are their surrogate decision-makers, and the pediatric nephrology team for taking decisions about KRT or conservative care. In this paper, we discuss the ethical challenges that arise relating to such decision-making, from a global perspective, using the framework of pediatric bioethics. While many ethical dilemmas in the care of children with KRT are universal, the most significant ethical dilemma is the inequitable access to KRT in low & middle income countries (LMICs) where rates of morbidity and mortality depend on the family's ability to pay. Children with kidney failure in LMICs have inadequate access to maintenance dialysis, timely kidney transplant and palliative care compared to their counterparts in high income countries. Using case vignettes, we highlight how these disparities place severe burdens on caregivers, resulting in difficult decision-making, and lead to moral distress among pediatric nephrologists. We conclude with key action points to change this status-quo, the most important being advocacy by the global pediatric nephrology community for better access to affordable kidney failure care for children.

## Introduction

“*Less than 10% of children with kidney failure worldwide actually receive dialysis or a transplant”* (1).

Kidney failure care consists of conservative treatment or kidney replacement therapy (KRT), i.e., maintenance dialysis or kidney transplant. Although dialysis is life-saving, the goal of successful pediatric KRT is a quick kidney transplant with either living or deceased donor organs.

Scholarship surrounding ethical challenges in kidney failure care in low resource settings has focused mainly on adult patients and does not apply well to children (2–5). Adults are considered responsible for their own treatment and are presumed to have the ability to make autonomous decisions including choosing conservative medical care only. There are well-developed conceptual models for adult conservative care and palliative interventions (6–9). In contrast, children lack autonomy to make medical decisions, are inherently vulnerable, and are dependent on their caregivers for decision-making and for treatment. Frameworks for palliative care are evolving but less well-developed (10). Although parents are considered responsible for their child, society shares responsibility for a child's well-being. Article 3 of the United Nations Convention on the rights of the child states that decision-making by parents, institutions or society should be with the best interests of the child as a primary consideration, yet, children live within families, communities, and societies each with limited resources (11).

The purpose of this paper is to describe the ethical challenges and disparities related to providing adequate kidney failure care for children from a global perspective, representing patients, caregivers and the healthcare team concerns. In low and lower middle income countries (LMICs), resource constraints are a major source of ethical dilemmas. Therefore, we will also discuss social determinants and injustices that frequently impact patient and family experience, and patient survival. We conclude with practical suggestions to address these ethical dilemmas.

## Principles of Medical Ethics and Decision-Making for Children with Kidney Failure

Medical ethics is frequently based on the framework of “Principlism” which holds that medical care should adhere to four principles: respect for autonomy, beneficence, non-maleficence, and justice to the greatest extent possible (12).

It is presumed that children lack decision making capacity and are unable to provide informed consent, therefore medical decisions must be made for children. Parents are generally considered surrogate decision-makers, and have the authority to provide informed consent for their child's treatment. In the case of kidney failure respect for autonomy may require clinicians to elicit the goals and values of the patient and their decision-maker and ensure that the decision-maker is adequately informed of appropriate treatment options.

The principle of beneficence requires seeking to provide care that would be expected to do the most good for the patient. Both parents and pediatric nephrologists have responsibility for the well-being of a child with kidney failure and are generally expected to make decisions in a child's best interest. The best interest standard requires decision-makers to weigh treatment choices by considering the present and future self-regarding interests of the child alone and choosing the option that maximizes benefit and minimizes harm for the child (13, 14). In the case of KRT beneficence may require decision-makers to weigh the benefits and harms of treatment options to the child and select the option that maximizes present and future benefit for the child and minimizes harm.

The principle of non-maleficence is commonly recognized as the duty to avoid preventable harms to the child. Like beneficence, both parents and pediatric nephrologists have duties of non-maleficence directed at children. Parents have such duties as a result of their position and responsibility for the well-being of the child. Pediatric nephrologists have such duties as a result of their clinical relationship with the child and their duty as citizen reporters and representatives of society. This is seen in child abuse and neglect laws which constrain parental decisions and recognize the interest of the state and society in children's well-being. Depending on country, parental decisions are constrained when they are not in a child's best interest (UK) or reach a threshold of clear, preventable, and imminent harm compared to an alternative (US) (15, 16). In the case of KRT non-maleficence may require clinicians to avoid treatment choices or to seek state intervention to prevent parental decisions that would be expected to result in preventable harm to the child.

Taken together the ethical frameworks of best interest or harm seek to highlight the primacy of the needs of the child, protect the child from exploitation by those more powerful, and hold that a child's basic needs must always be met (13–15).

The principle of justice has multiple meanings at the levels of patient care, health systems, and society, but is fundamentally concerned with fair, equitable, and appropriate treatment in light of what is owed to persons (12). A minimal requirement of justice in bioethics is traditionally reflected in Aristotle's formal principle of justice which holds equals must be treated equally, and unequals treated unequally (12). In the case of kidney failure care justice may require decision-makers considering treatment choices for two children with kidney failure be treated similarly unless there are morally meaningful differences between them. Factors such as family resources and wealth are not generally considered as morally meaningful differences.

Although the four principles are considered to be of equal value, in the clinical setting of pediatric kidney failure, they may conflict. KRT is burdensome on children and their caregivers and is expensive and must be paid for. Parents and healthcare providers may disagree on the perceived benefits and harms of dialysis or transplant and parents may refuse KRT treatments. Efficacious dialysis and transplant are clearly in the best interests of a child with kidney failure regardless of where they are in the world. However, a child always exists in the context of their family, community, and society. The “best choice” may not be clear or the path to pursue it may be difficult or impossible. In LIMCs these ethical challenges both inform and are compounded by the disparities experienced by children with kidney failure, their families, and their clinicians.

## Disparities in Kidney Failure Between HIC and LMICs

The incidence of pediatric kidney failure treated with KRT from HIC ranges from 9–15 per million age-related population (pmarp) (17–20). Similar data from LMICs is very limited due to the lack of registries, and is usually based on single center studies. In South-East Asia and Sub-Saharan Africa where maintenance dialysis is not available to the majority, the reported incidence of pediatric kidney failure is very low (<10 pmarp) (1, 21–23).

In HIC, congenital anomalies are the leading cause of kidney failure. In LMICs chronic glomerulonephritis has been more frequently reported than congenital anomalies. Regardless of the native kidney disease, chronic kidney disease (CKD) in LMICs is diagnosed later, progresses faster to kidney failure and is associated with more comorbidities than in HIC (24–26). In LMICs with a colonial legacy, the effects of historic societal injustices play a role in pediatric kidney disease. Poor sanitation, lack of access to clean water and polluted environments are a source of frequent infections that can cause glomerular disease, acute kidney injury (AKI) and ultimately progression of CKD (27). Limited access to perinatal care, low birth weight and poor primary healthcare result in late diagnosis, incomplete evaluation and poor follow up of children with congenital anomalies. Due to the lack of awareness of CKD symptoms, opportunities for preventative and disease modifying care are lost (28). Referral to a pediatric nephrology center occurs late, when the child is already in kidney failure and the decision to initiate dialysis is taken in a crisis situation (29–31).

Thus there are inequities in the rates of acquired kidney disease and availability of primary and secondary level CKD care. It is important for the global pediatric nephrology community to be cognizant of these differences because they bring to light important ethical dilemmas that nephrologists in low resource settings encounter when approaching decision-making for a child with kidney failure. Treating kidney failure patients in such circumstances is difficult and frequently disheartening. These disparities, described in more detail below, underscore the need for advocacy from the global nephrology community to support the needs of children with kidney disease, their families as well as the healthcare teams that care for them.

## Inequity in Access to Dialysis

There is a wide disparity in the availability of pediatric dialysis (32, 33). In high income countries (HIC) pediatric dialysis is easily accessible and financially supported by the government. While over 50% of the world's children live in LMICs, maintenance dialysis for children is virtually non-existent in Sub-Saharan Africa (21) and very limited in India and other regions (23, 34). In LMICs there is inadequate maintenance dialysis infrastructure and insufficient numbers of pediatric nephrologists for the population served (35). There is a lack of universal healthcare coverage for dialysis in most LMICs. This is mainly due to inadequate public healthcare expenditure on pediatric dialysis (17, 36, 37). Thus, the availability of KRT does not necessarily imply that these services are accessible to patients. Free or subsidized adult services may be available, but pediatric dialysis is rarely accessible. Even when government supported dialysis is available, rationing of dialysis by the healthcare system frequently occurs due to the shortage of hemodialysis (HD) beds or peritoneal dialysis (PD) dialysate bags. This results in intentional under-dialyzing, with poor quality of care overall. In South Africa, provision of dialysis may be limited those who agree to pursue transplant, reducing the opportunity for voluntary decision-making (38, 39).

In the private sector, where the majority of pediatric dialysis occurs, treatments are expensive, with high out-of-pocket (OOP) costs to families resulting in catastrophic healthcare expenditure (40, 41). Further, utilization of dialysis is impacted by need for travel to access care (23, 42). Treatment non-adherence is common and dialysis is commonly discontinued resulting in high rates of patient death or loss to follow up (29, 43–45). The net effect is significant patient suffering, family suffering, and clinician suffering with little benefit and incredible expense—not because dialysis treatment does not or cannot work, but because the resources needed for it are not available.

## Inequity in Dialysis Experience and Outcomes

Inequity in the experience of caregivers of children with kidney failure in LMIC contributes to disparities in patient survival and the experience of pediatric nephrologists. Caregivers of children with kidney failure experience a sustained and heavy burden of care in both HIC and LMICs. Caregiver burden is defined as the perception of “overload” experienced by an individual in the physical, emotional, social and financial domains, as a result of the caregiving process (46–48).

In addition to the emotional distress at their child's diagnosis of an irreversible life-limiting disease, caregivers must assume responsibility for medical care. Globally, caregivers of children on dialysis face physical stress without respite, have no time for self-care and have reported chronic pain, weight gain, hypertension and diabetes (49–52). Mental illnesses such as depression, anxiety and medical traumatic stress are also prevalent (53, 54). Caregivers of children on dialysis (especially PD) experience social isolation and difficulty in caring for other children (55). Even in HIC, families of children with kidney failure face significant financial burdens from travel costs and loss of income due to the inability of the primary caregiver to work outside the home (56).

In LMICs these burdens are often magnified, particularly financial costs. Caregivers experience more severe financial hardship, especially those paying OOP for dialysis. The monthly costs of dialysis are frequently higher than the family's income resulting in catastrophic healthcare expenditure (57–60). Caregivers resort to selling personal property or taking loans to support payment for dialysis (61, 62). Peritoneal dialysis is the preferred dialysis modality in young children and in those without access to an outpatient HD center. However, PD is more expensive due to the higher cost of consumables. Caregivers in LMICs are expected to do manual PD exchanges for 12–16 h a day as automated PD is too expensive, or unreliable due to irregular power supply (63, 64). In addition to financial stress, caregivers experience physical exhaustion and social isolation. Kidney failure may be considered a social stigma by the child's family, community or even by a parent, denying support to the child and the primary caregiver.

Single center studies of kidney failure from LMICs show very poor patient outcomes. When families are unable to meet costs, they reduce the number of PD cycles or days on HD. Due to routine under-dialysis, children have higher rates of infectious complications, more morbidity and poorer quality of life (QoL) compared to their HIC counterparts (65, 66). Death or discontinuation of treatment occurs in 50–80% of children (29, 67). The stigma surrounding foregoing dialysis can prevent opportunities for children to receive compassionate conservative care or symptom focused palliative treatment, adding further suffering.

Pediatric nephrologists in LMICs work with children and families doubly burdened by disease and limited resources and must accept inadequate dialysis, fewer investigations than ideal, infrequent patient follow up visits and treatment refusal by caregivers. Constant compromise of quality of care results in physician distress and frustration. Moral distress occurs when clinicians know the ethically correct action to take but are constrained from taking this action (4, 68–70). Healthcare providers may see themselves as unethical when treatment for kidney failure is incomplete or a child or caregiver experiences suffering (71). Physicians also experience moral distress at being unable to help families who cannot pay for treatment. Ultimately, moral distress can take an emotional toll on physician well-being, threaten core values, and lead to burnout (72).

In sum, in LMICs limited dialysis availability and resources to support treatment contribute to greater burdens and poorer outcomes experienced by children with kidney failure, their families and their clinicians.

## Disparities in Kidney Transplant Between HIC and LMICs

A kidney transplant is the optimal treatment for children with kidney failure offering an opportunity for better lifespan, growth and development and QoL compared to dialysis (73). Living donor transplant is superior to deceased donor transplant in terms of graft survival, but relies on the availability of an evaluated, compatible and willing donor. Deceased donor kidney transplant depends on the availability of deceased donor organs, a well-organized national or regional organ sharing network and ethical, transparent allocation policies. There is a wide global disparity in the availability of adult kidney transplant centers with LMICs experiencing much lower rates of transplant than HIC (36, 74). These disparities are even greater for pediatric kidney transplant.

## Inequities in Access to Kidney Transplant

Pediatric kidney transplant centers in LMICs successfully perform transplants, with excellent short term graft survival but access to transplant and transplant centers remains limited (75–79). There are insufficient pediatric transplant centers and transplant rates for Sub-Saharan Africa and South East Asia remain absent or dismally low (80–82). Although cost-effective compared to dialysis, transplant is expensive and not a priority for government healthcare spending (83, 84). The centers that do offer pediatric transplant services are mainly in the private sector with prohibitive OOP costs. Thus, inability to pay is an insurmountable barrier excluding economically disadvantaged families from transplant.

The majority of transplants in LMICs are from living donors (81, 85). There is insufficient investment in national deceased donor organ procurement and organ allocation networks in LMICs like India. Organ allocation practices vary globally, and frequently within different regions of the same country (39, 40, 86). Most listing criteria in low resource countries require that kidney failure patients (including children) receive dialysis for at least 3 months prior to being activated on the wait list. This results in lower rates of pre-emptive transplant with its superior outcomes (87).

After blood group compatibility, wait time on the list is the deciding criteria for organ allocation in most LMIC (88, 89). In contrast organ sharing networks in HIC prioritize children over adult kidney failure patients on the wait list (86, 90). Pediatric prioritization shortens wait time and often provides access to better “quality” organs. In low resource settings many organ allocation policies do not prioritize children, resulting in prolonged wait times. Some have argued that prioritizing children when demand is vastly greater than supply goes against ethical principles (91). On the other hand, Table 1 summarizes arguments in favor of prioritizing children while adhering to the key principles of bioethics. Cultural belief in the importance of prioritizing earning adults who must support families over children may also play a role in allocation schema. While such beliefs may be common in some communities/societies, these policy decisions are usually unilateral with no stakeholder participation.

**Table 1:** Ethical principles supporting pediatric prioritization in deceased donor kidney allocation.

**Bioethics principle**	**Rationale for pediatric prioritization**
Beneficence	• Better survival, QoL, lower morbidity than dialysis • Opportunity to grow, and overcome the physical and neurocognitive disabilities unique to pediatric CKD
Justice	• Children deserve a “fair innings” at enjoying medical well-being as they grow, as all individuals deserve the opportunity to live through each life-stage
Utility	• Children have less comorbidities and are expected to survive longer than an older adults with kidney failure
Equity	• Prioritizing children balances the advantage that adults have over children in terms of waitlisted time • Recognizes that children do not bear responsibility for organ failure and are an inherently vulnerable group
Non-maleficence	• Rarity of pediatric kidney failure ensures that a very small number of kidneys get diverted away from waitlisted adults

*QoL, quality of life; CKD, chronic kidney disease*.

The consequence of limited organ allocation infrastructure and lack of pediatric prioritization means that in LMIC almost all transplants are from living donors, yet barriers to living donation remain.

## Barriers to Living Donor Kidney Transplant

Living kidney donation is the highest form of altruism, yet donors face significant financial burdens. In HIC despite policies to reimburse living donors for medical expenses donation is still associated with significant indirect costs from travel expenses and loss of income from unpaid leave (92–94). Low income groups, racial and ethnic minorities face a disproportionately greater financial burden from living donation, potentially explaining lower pre-emptive and living donor transplant rates observed for children from lower socioeconomic status (88, 95, 96).

In LMICs living donor evaluation places even greater burdens on the donor family. In accordance with the Declaration of Istanbul, monetary compensation for organ donation is strictly forbidden by law (97). For example, in India, although altruistic donation may be technically legal, the burden of proof of absence of transplant commercialism is laid on healthcare team. To avoid legal tangles, most centers restrict living donors to immediate family members (parents and grandparents for a pediatric recipient) (80, 84, 89). Living donors do not receive any form of reimbursement for medical expenses, lost wages or travel expenses. Thus caregivers face the double expense of paying for dialysis as well as donor evaluation. Those earning daily wages risk losing their jobs if they take a prolonged absence from work. Living donation is more feasible when caregivers have salaried jobs and paid leave. Thus, a family's socioeconomic status often determines whether the child has access to a living donor transplant.

Women compose the majority of kidney donors worldwide (29, 74, 98). Living kidney donation is associated with surgical risk and the possibility of CKD in the long-term. Families with a single earner, usually the father, may assume that the child's mother would volunteer to donate (99, 100). It has been argued that parents do not view their own interests as separate from their child's negating concerns about coercion (101). However, mothers may be unable to refuse, from a sense of family obligation and emotional and financial dependence, indicating a lack of choice. Alternatively, mothers may be prevented from donating as families consider her the primary caregiver of the child and also the family (102). Whether as an organ donor or restricted to a role of care labor, women may be silent victims of exploitation.

With the reduced access and significant barriers to obtaining transplant in LMICs children and families are forced to continue dialysis. Children who remain on dialysis have higher mortality than those on transplant and some will face certain mortality when accumulated debt from dialysis treatment (which they must also pay for) becomes too great and treatment is forgone.

## Inequities in Transplant Experience and Outcomes

There is very limited registry data on graft survival from LMICs. In single center, small studies long term transplant outcomes are not as good at HIC. Unfortunately, patient mortality (with a functioning graft) occurs and is usually due to serious infections or sepsis (77, 103). Although short term graft outcomes are compatible with HIC, allograft survival at 5 years were consistently <80% and much lower at 10 years, in spite of the predominance of living donor transplants (81). The most frequently cited causes for graft loss were chronic rejection and infections (75, 78, 104). Infrastructure for immunosuppression drug level monitoring, histopathology and viral load surveillance is underdeveloped in most LMICs. Although generic drugs are available at a lower cost than in HIC, these may be inconsistent in potency (105). Drug level monitoring is expensive and performed less frequently than ideal. Ability to pay for post-transplant care, continued immunosuppression and laboratory monitoring remains a major concern in LMIC and a possible cause for graft failure. Second transplants are rarely achieved.

## Ethical Dilemmas in Decision-Making in KRT

## Parental Refusal of Dialysis

Caregivers may refuse to initiate dialysis because they consider the therapy too burdensome for their child, themselves or their families. This case highlights the difference in outcomes in HIC and LMICs when parents refuse dialysis. Traditionally, in Western bioethics, pediatric medical decision-making is centered around “best interest” of the child alone. As life-saving therapy, dialysis may be “morally obligatory” for children with kidney failure in most HIC countries and caregiver refusal considered neglectful, potentially resulting in child endangerment (106, 107). While social safety nets are imperfect even in HIC, professional guidelines for dialysis decision-making consider differing family values and family burdens to varying degrees, but stop short of allowing dialysis to be withheld unless severe comorbidities existed (108, 109).

In LMICs decision-making occurs within the context of the resources available. Where there are inadequate resources for society to pay for dialysis, state intervention would be unfeasible and parents have the final authority over KRT decisions. Refusal due to financial constraints may not represent a free “choice” (4). Regardless of the reason for refusal, physicians in LMICs may have no legal recourse to enforce initiation of dialysis for a child. For example, in India, if parents of a child <12 years of age refuse dialysis, physicians cannot proceed against the parents' wishes even though the treatment is considered life-saving (110). For older children, appealing the judicial system may be theoretically possible, yet in the absence of a functional social welfare system, physicians will be unable to achieve adequate treatment.

An unfortunate consequence of foregoing dialysis is the termination of the physician-family relationship. The family is afraid to return for fear of being blamed for non-adherence. This results in additional harm to patients and families as they are denied benefits of symptom alleviation from palliative care. The goal of pediatric palliative care in kidney failure is to improve the QoL of children and families, continuing concurrently with, or in the absence of, other KRT modalities. The key areas of focus are symptom alleviation, social support, mental health and communicating with the family about patient centered outcomes. Thus while the benefits of palliative care extend beyond end-of-life care and hospice, they are especially important in LMICs when KRT is not accessible or associated with heavy burden.

## Clinician Decision-Making in LMICs

The fundamental difference between caring for children with kidney failure in HIC and LMIC is not a difference in ethics but a lack of accessible KRT therapies or sufficient resources to carry them out effectively. In HIC with universal healthcare coverage and KRT infrastructure, the decision to pursue dialysis or transplant for a child with kidney failure rarely presents an ethical dilemma. Such treatment is clearly in the best interest of a child with kidney failure except in the rare situations when KRT would not be expected to improve the child's QoL or survival. In LMICs also, dialysis and transplant would clearly be in the best interest of a child with kidney failure and foregoing such therapy would result in imminent harm. However, healthcare providers in LMICs encounter unique ethical challenges in advising families of children with kidney failure when KRT is either unavailable or associated with very high burdens or OOP costs.

Physicians frequently lack the training to approach such difficult decision-making and avoid such conversations. The consequence may be clinicians limiting their involvement to a presentation of choices (however limited) and subsequently withdrawing from the decision-making process leaving patients and parents isolated and without guidance. Alternatively, clinicians may make a unilateral decision that pursuing KRT is futile because there is no clear mechanism to pay for it, especially when the patient and family are extremely poor. Information about treatment options may be withheld in the belief that caregivers are best spared the distress of learning that dialysis and transplant are inaccessible. Such paternalism denies the family an opportunity to make a truly informed choice and potentially the chance to pursue treatment elsewhere (111).

In other instances parents may be overwhelmed by a diagnosis of kidney failure. It has been suggested that physicians refrain from advising families to pursue or forego dialysis and instead encourage autonomous decision-making after educating the family the about their choices. However, in reality, pediatric nephrologists actively participate in decision-making regarding KRT. While models of shared decision-making highlight the importance of clinicians providing education and ascertaining goals and values from the patient and family as steps toward reaching a decision, families are frequently overwhelmed with the new diagnosis of kidney failure. They do not feel empowered to make complex medical decisions and may heavily rely on their pediatric nephrologist to guide decision-making. In these situations, the pediatric nephrologists' decision to offer or recommend dialysis may be closer to determinative. In those circumstances, the doctor has an additional responsibility of prioritizing the patient's needs while abiding by ethical values and norms.

Ethical guidance in these cases can be conflicting. Where resources are limited and caregiving burdens are significant, it is possible, and even likely, that a treatment choice may be in the best interest of a child, but not in the best interest of other children in the family, the family, or the community. A strict interpretation of the best interest standard limits considerations to the present and future self-regarding interests of the child alone. As previously discussed, by this calculus, efficacious KRT is clearly in a child's best interest, but this overlooks the impacts of the expense and burdens of the treatment placed on other family members and ultimately the child herself if money runs out. Further, the best interest of the child alone overlooks that parents, siblings, and other caregivers are also moral agents with finite resources and pragmatically that even in the wealthiest countries public resources may be insufficient to meet the needs of children with kidney disease and their families. To address this tension, some scholars have attempted to develop a “greater best interest of the child” recognizing not only the interests of the child, but those of parents, extended family members, along with cultural and social issues (112, 113). The philosopher Loretta Kopelman proposed a case of a treatment which could extend a child's life by one day, but would bankrupt the family due to its expense. While the treatment would benefit the child, Kopelman holds that the best interest standard could not support such an option as the interests of others place limits of “reasonability” on which choices should be considered (114). While extreme in nature, Kopelman's case is not dramatically different than situations pediatric nephrologists practicing in LMIC find themselves. Severe harms to others must be taken into account even if they are left out of a strict interpretation of best interests of the child. This raises further concerns of fairness, equity, and justice.

## Fairness, Equity, and Justice

Fairness dictates that we avoid making treatment decisions on morally irrelevant factors. Disease and the expense of treating it are not distributed equally, nor are children generally viewed as being responsible for their state of health. Ability to pay for treatment is traditionally viewed as not morally relevant. If medical care is a basic necessity then excessively high costs of KRT are unjust because they exploit the vulnerability of the patient and their family. As a consequence, fairness may dictate a just society to support mitigating the expense of medical care for children and the demands such care place on others. Although intuitively appealing, this interpretation of justice is challenged by realities encountered by pediatric nephrologists including expensive treatments, impact on caregivers and families, and systemic and societal injustices among others. These challenges impact individual patient encounters, work with communities and within a healthcare system, and the role of nephrologists as global citizens.

We do not live in just societies which meet the needs of each child. Resources and opportunity are not equally or equitably distributed. This can be a source of moral distress for patients, families, and clinicians as decisions or treatment options may not match what ethics or fairness or the child's best interest seems to demand. There can be certainty about the most approriate course for the child, yet it cannot be pursued. This injustice is a powerful call for advocacy from the global nephrology community to increase the availability of resources for children with kidney disease, but also highlights the need for clinicians and ethicists to engage with patients and their families within the society/environment in which they find themselves.

Non-ideal theory of justice recognizes that we live in an imperfect society. In this theory, justice requires us to work to progress toward a more ideal state, but also engage in the present. A key feature of non-ideal theory is that even if things are not fair, the burdens of unfairness should not fall exclusively on those most vulnerable such as children with kidney failure (115). Alternatively it would also be wrong for the full burdens of society's failures to fall on parents of a child with kidney failure, although perhaps less wrong than solely on a child. This framing again highlights the need for communities, societies, and the global nephrology community to assume some of the burden and responsibility for the well-being of children with kidney failure and their families.

## Suggested Action-Points for Resolving Ethical Challenges in Pediatric KRT

**Figure 1:**
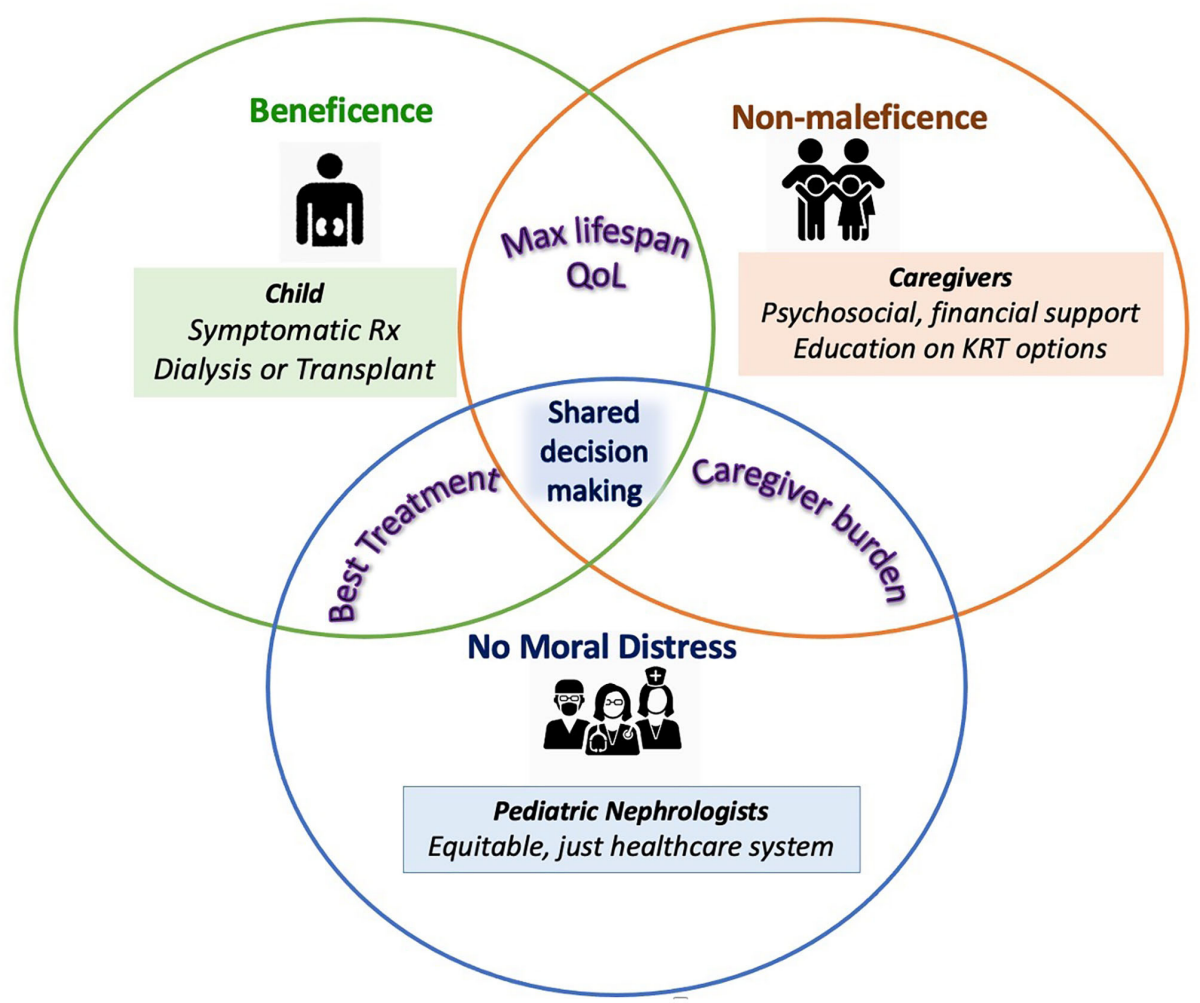
Ethical considerations in kidney failure care for children. Each circle represent stakeholders in pediatric kidney failure care—the patient, caregivers and the healthcare team with their key clinical and ethical needs in colored text and boxes. The key interests of each stakeholder with regards to the other are represented in the overlap of their respective circles. Potential targets to support achieving these interests and overcoming ethical challenges are listed in each stakeholder's domain. As a central clinical and ethical requirement, shared decision-making is at the center of pediatric kidney failure care.

The clinical and ethical challenges encountered in the care of children with kidney failure are multidimensional and complex (Figure 1). Nevertheless it is the duty of the global nephrology community to take steps to address them. Pediatric nephrologists worldwide must accept a broader responsibility while caring for children with kidney failure. These include support for primary and preventative care, advocacy for patients with kidney failure and their families and a better focus on palliative care. The action-points suggested have been classified as “Short-term strategies for change” and those with a longer horizon as “Longer-term Goals.” It is our hope that addressing these action items may help to address the concerns highlighted in Cases 1–3.

Case 1: A 10 year old boy in India presented to a referral pediatric nephrology center in kidney failure. His parents were daily wage laborers, living 250 km from the center. Government supported dialysis was not available in the area and the family paid out of pocket to initiate manual CAPD. After discharge, his mother stopped working to perform PD and provide medical care. They had difficulty paying for dialysis bags and resorted to selling the family property and skipping dialysis a few days a week. Clinic visits were also irregular as the parents could not afford the costs of travel and lab tests. This resulted in a PICU admission for hypertension and fluid overload, and the family faced further expenditure. After discharge, the patient was lost to follow up and the family could not be contacted. Three months later, a relative called the medical center to say that the child had died due to "breathing difficulty" two weeks after his parents had discontinued dialysis altogether.

Case 2: An 11 year old boy with kidney failure due to FSGS has been on the deceased donor wait-list for 5 years without receiving a transplant. He has no compatible living donors and paired exchange, or ABO incompatible transplants are not accessible. National transplant policies do not allow unrelated living organ donation. The deceased donor transplant rates in the country are very low and there is no organized national organ sharing network. The regional organ allocation program where he is listed does not prioritize children in any way. Thus, his parents have borne the out-of-pocket expenses for transplant evaluation, continued dialysis, and an unrelenting burden of care. The child has multiple progressive comorbidities associated with kidney failure including severe HTN, LVH and CKD MBD. He is too unwell to attend school regularly or play with friends.

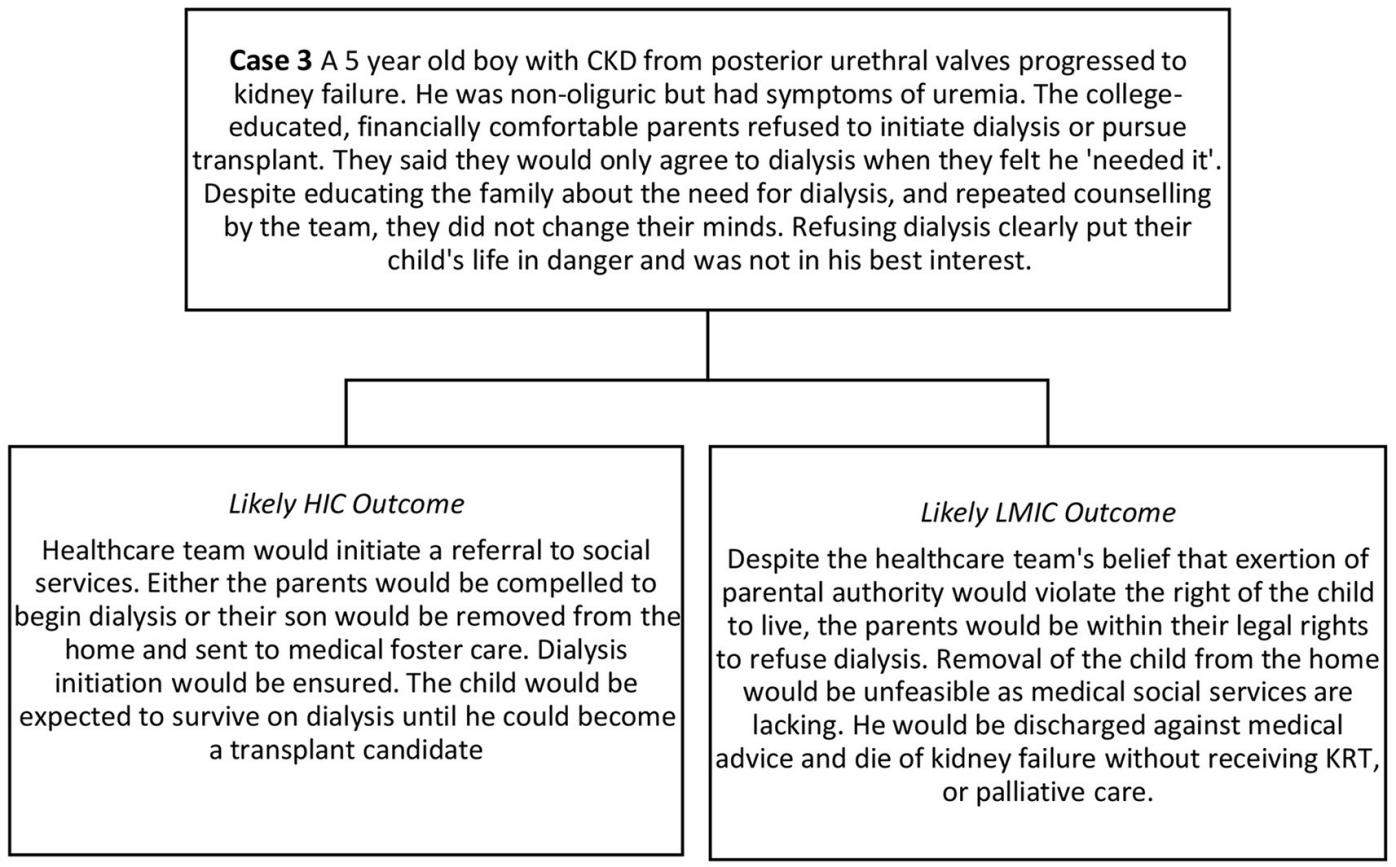

### Short-Term Strategies for Change

1. *Recognizing the strengths of children with kidney failure and their parents*. As discussed above children with kidney failure and their families face extraordinary burdens, particularly in LMIC. The disparities experienced among children with kidney failure in LMIC are troubling and demand the attention of the global nephrology community, yet it would be wrong to cast these children and their families solely or largely as victims. This overlooks their strengths. In spite of barriers, many children with kidney failure are able to thrive, continue their education and are valued members of families and communities. Parents and families can and do go to extraordinary lengths to provide needed care for children with kidney disease demonstrating love, ingenuity, resourcefulness, and skill balancing their child's medical needs with other duties. These are strengths that deserve acknowledgment and respect.
2. *Utilizing procedural justice*. Denial of access to medical treatment based on ability to pay raises serious concerns of justice. In the setting of unavoidable resource shortage it is necessary to develop clear, explicit procedures for considering decision-making for every child (65, 116, 117). These procedures must be country or region specific, informed by relevant evidence and ethical principles and take into account available resources and social supports. Decision making should involve all stakeholders (patients, caregivers, nephrologists, community leaders) and the process should meet 4 conditions for fairness: publicity, relevance, revision and appeal, and regulation (117).
3. *Duty to care and the importance of palliative care*. Despite the natural focus on optimal treatment for the child, pediatric nephrologists should be conscious of the financial and psychosocial burdens of KRT and understand how these might play a role in family decision-making. For all children facing kidney failure treatment options including dialysis and transplant should be discussed with the child and the family, even if the availability of those modalities is limited or are associated with dramatic out of pocket costs. However, caregiver counseling must take into account each family's unique circumstances, to enable shared decision-making. In resource constrained setting, families may opt for conservative care only. Physicians continue to have a duty to care for these patients, despite incomplete treatment and accept family-prioritized outcomes such as maximizing QoL. Additionally, pediatric nephrologists need better awareness of palliative care to address kidney failure symptoms of those who are on dialysis and those on conservative care (109). The goal of palliative care is not focused on “end-of-life” but to add “more life to a patient's years” (10, 118).

### Longer-Term Goals

1. *Addressing primary healthcare needs and improving awareness of pediatric kidney disease to increase early detection and referral for treatment*

Supporting societal efforts to promote health for all and facilitate primary healthcare would facilitate awareness of CKD, early diagnosis and referral. If detected early, the progression of CKD in children can be slowed. Similarly, measures that improve sanitation and access to clean water must be supported to prevent AKI episodes (119).

Recognition of kidney disease as an important non-communicable disease by international agencies funding child-health such as the World Health Organization would place a spotlight on CKD awareness. Training primary healthcare workers to recognize signs of pediatric kidney disease would improve early diagnosis and referral for treatment, especially in rural, underserved areas. Educating the community about the causes and treatments available for pediatric kidney disease will help reduce the stigma of CKD.

2. *Need for advocacy for children with kidney disease, their families, and their nephrologists*

To improve survival and QoL of children with kidney failure and to reduce physician moral distress, the global pediatric nephrology community needs to advocate for better pediatric KRT access globally. Healthcare programs must improve the awareness, availability, accessibility, and affordability of dialysis and transplant (120). Important areas of focus include universal healthcare coverage for pediatric dialysis and transplant organ allocation policies that prioritize children on the wait list. Although charitable donations might represent a life-line for individual patient families who are unable to afford care, public funding for pediatric KRT is the more sustainable solution to reduce catastrophic healthcare expenditure and impoverishment (42, 58).

3. *Utilization of international nephrology society initiatives to achieve sustainable improvements in kidney failure care*

Current efforts to improve kidney failure care in LMICs include training of pediatric nephrologists and allied professionals in partnerships funded by international nephrology and transplant societies. Examples of such collaborative training include the International Society of Nephrology (ISN) and International Pediatric Nephrology Association (IPNA) Sister Renal Centre programs and the outreach program by International Pediatric Transplant Association (IPTA) to facilitate pediatric dialysis and transplant programs. The IPNA World Kidney Day program funds initiatives to improve primary and secondary prevention strategies for pediatric CKD. The Saving Young Lives initiative, jointly supported by the ISN, IPNA, the International Society for Peritoneal Dialysis and European Peritoneal Dialysis, works with hospitals in low resource settings to establish acute PD services. These important efforts face a challenge to ensure that advances in care through short-term partnerships result in sustainable improvements in kidney failure care.

## Conclusions

Children with kidney failure suffer from a chronic disease requiring lifelong treatment. Pediatric kidney failure care is associated with multiple ethical challenges from the perspective of the patient, caregivers and healthcare system. Yet, the most significant ethical dilemma is the inequitable access to KRT in LMICs where rates of morbidity and mortality depend on the family's ability to pay. Advocacy efforts from the global pediatric nephrology community are imperative for resolving these ethical dilemmas.

## Author Contributions

PP and AW contributed equally toward the conceptualization, writing, editing, and finalizing the completed manuscript. Both authors contributed to the article and approved the submitted version.

## Conflict of Interest

The authors declare that the research was conducted in the absence of any commercial or financial relationships that could be construed as a potential conflict of interest.

## Publisher's Note

All claims expressed in this article are solely those of the authors and do not necessarily represent those of their affiliated organizations, or those of the publisher, the editors and the reviewers. Any product that may be evaluated in this article, or claim that may be made by its manufacturer, is not guaranteed or endorsed by the publisher.
